# α2,6 sialylation distinguishes a novel active state in CD4^+^ and CD8^+^ cells during acute *Toxoplasma gondii* infection

**DOI:** 10.3389/fimmu.2024.1429302

**Published:** 2024-08-26

**Authors:** Diego Sierra-Ulloa, Jacquelina Fernández, María Cacelín, Gloria A. González-Aguilar, Rafael Saavedra, Eda P. Tenorio

**Affiliations:** ^1^ Departamento de Bioquímica, Facultad de Medicina, Universidad Nacional Autónoma de México, Mexico City, Mexico; ^2^ Departamento de Inmunología, Instituto de Investigaciones Biomédicas, Universidad Nacional Autónoma de México, Mexico City, Mexico; ^3^ Posgrado en Ciencias Biológicas, Universidad Nacional Autónoma de México, Mexico City, Mexico

**Keywords:** T lymphocytes, *Toxoplasma gondii*, α2, 6 sialylation, T cell functional states, *Sambucus nigra*, flow cytometry

## Abstract

Toxoplasmosis is a worldwide parasitosis that is usually asymptomatic; cell-mediated immunity, particularly T cells, is a crucial mediator of the immune response against this parasite. Membrane protein expression has been studied for a long time in T lymphocytes, providing vital information to determine functional checkpoints. However, less is known about the role of post-translational modifications in T cell function. Glycosylation plays essential roles during maturation and function; particularly, sialic acid modulation is determinant for accurate T cell regulation of processes like adhesion, cell-cell communication, and apoptosis induction. Despite its importance, the role of T cell sialylation during infection remains unclear. Herein, we aimed to evaluate whether different membrane sialylation motifs are modified in T cells during acute *Toxoplasma gondii* infection using different lectins. To this end, BALB/c Foxp3^EGFP^ mice were infected with *T. gondii*, and on days 3, 7, and 10 post-infection, splenocytes were obtained to analyze conventional (Foxp3^-^) CD4^+^ and CD8^+^ populations by flow cytometry. Among the different lectins used for analysis, only *Sambucus nigra* lectin, which detects sialic acid α2,6 linkages, revealed two distinctive populations (SN^Bright^ and SN^-/Dim^) after infection. Further characterization of CD4^+^ and CD8^+^ SN^-/Dim^ lymphocytes showed that these are highly activated cells, with a TEf/EM or TCM phenotype that produce high IFN-γ levels, a previously undescribed cell state. This work demonstrates that glycan membrane analysis in T cells reveals previously overlooked functional states by evaluating only protein expression.

## Introduction

Toxoplasmosis is a worldwide parasitosis caused by the apicomplexan parasite *Toxoplasma gondii.* It is estimated that 30% of the population is infected, but most individuals remain asymptomatic throughout their lives. Infection during pregnancy may cause malformations or abortion, and reactivation due to immunosuppression can lead to encephalitis or death. The immune response against *T. gondii* has been widely studied, demonstrating that cell-mediated immunity is essential for infection control. This parasite induces high amounts of IL-12, TNF-α, IL-1, and IFN-γ produced by dendritic cells, macrophages, type 1 ILCs, and NK cells, which lead to a T_H1_ polarized response with INF-γ-CD4^+^ producing T cells that generate a vigorous CD8^+^ cytotoxic activity (1–4).

Analysis of T cell membrane proteins has allowed us to understand and associate particular expression profiles to specific phenotypes and cell processes like maturation, activation, cell polarization, exhaustion, memory generation, etc (5, 6). Studying these molecular fingerprints and their dynamics has provided essential clues to comprehend T cell biology better and promote immunotherapy design (7, 8). However, recent information obtained from single-cell analysis and transcriptomics has redefined our understanding of T cells. The amount of information obtained using this approach demonstrates that T cell plasticity could lead to an unimaginable amount of subsets and fates depending on the analyzed context and timing (9–13). This leads us to consider the inclusion of terms like cell types, fates, and/or states when talking about cell stability, function, and temporality rather than limiting our descriptions of different populations defined by a single phenotype (13).

Further complexity is revealed after recalling that T cell function is not solely related to protein expression; posttranslational modifications, like glycosylation, are essential for T cell activation and have been associated with different maturation and functional stages (14–16). For example, in the thymus, *O*-glycan and *N*-glycan α2,6 sialylation occur during T cell maturation (17–20). In mature cells, previous studies showed that after activation, mouse T cells switch the expression of *N*-glycolylneuraminic acid to *N*-acetylneuraminic acid, followed by a sialic acid (Sial) linkage change from α2,6 to α2,3 (21). *O*-glycan biosynthesis in T cells changes after activation (22, 23); activated CD4^+^ T cells show a reduced expression of Sial α2,6 and Sial α2,3 while increasing the Sial α2,8 linkage (24); and *in vitro* polarized T_H1_ and T_H2_ human CD4^+^ T cells present different glycophenotypes, this is, T_H1_ but not T_H2_ cells express less Sial α2,6 molecules and higher levels of asialylated Core-1 *O-*GalNAc glycans, a modification related to apoptosis regulated by Galectin 3 (25).

However, little is known about the temporal dynamics of lymphocyte glycosylation during infection. Thus, this work aimed to analyze CD4^+^ and CD8^+^ glycophenotypes using a *T. gondii* acute infection mouse model. To this end, we first used *Sambucus nigra* (SN) and *Maackia amurensis* lectin II (MAL II) to analyze α2,6 and α2,3 sialylated molecules (Sial α2,6 and Sial α2,3), respectively, and Peanut agglutinin (PNA) along with *Amaranthus leucocarpus* lectin (ALL) to determine asialylated and sialylated/asialylated core-1 *O*-GalNAc glycans, respectively. Given that only SN revealed two distinctive populations after infection (SN^Bright^ and SN^-/Dim^), we further characterized them within CD4^+^ and CD8^+^ cells. We analyzed Sial α2,6 coexpression with CD69 and CD25 to evaluate its possible relation with activation progression (7, 26) and determined IL-2, IFN-γ, and IL-10 production in each glycophenotype. Using CD44 and CD62L, we determined if these SN^Bright^ and SN^-/Dim^ cells belong to a naïve (TN), effector/memory (TEf/EM), central memory (TCM), or terminally differentiated effector memory (TEMRA) cell populations (27, 28). Finally, we propose that combining analysis of glycan, lineage, activation, and differentiation phenotypes provides a new perspective to describe T cell functional states.

## Materials and methods

### Mice

Six–eight-week-old BALB/c Foxp3^EGFP^ knock-in and Swiss-Webster mice were bred at Instituto de Investigaciones Biomédicas Animal House and kept in micro isolator cages according to local guidelines. All experiments were performed using age and sex-matched animals, and protocols were approved by the Institutional Bioethics Committee for Animal Research.

### Parasites and infection

The ME49 strain of *T. gondii* was used for all experiments. Parasites were maintained in Swiss-Webster mice by i.p. infecting 10 cysts obtained from the brains of infected mice as previously described (29). For peroral experimental infection, mice were anesthetized with Sevorane (Abbott) and infected by gavage with 10 cysts obtained from Swiss-Webster mice infected 2-4 months earlier.

### Splenocytes isolation

After 3-, 7- and 10 days post-infection, animals were euthanized, spleens were removed and cells were obtained by perfusion with Dulbecco’s phosphate buffer saline (DPBS). Erythrocytes were lysed with a hypotonic NH_4_Cl solution. Splenocytes were washed, resuspended with DPBS, and used immediately.

### Flow cytometry, antibodies, lectins, and reagents

Monoclonal antibodies used for flow cytometry analysis were obtained from Miltenyi: anti-CD4 PE-Vio770 (REA604), -CD8 PE-Vio770 (REA601), -CD25 PE (REA568), and -CD69 PerCP-Vio700 (REA937), -CD44 PE (REA664), -CD62L PerCP-Vio700 (REA828), -CD16/CD32 Vio Bright FITC (REA377); from Biolegend: anti-CD4 APC-Cy7 (RM 4-5), -CD25 Brilliant Violet 421 (PC61) -IFN-γ PE-Cy7 (XMG1.2), and -IL-10 PE (JES5-16E3) or from eBioscience anti- IL2 eFluor 450 (JES6-5H4) or from Tonbo Biosciences anti-CD8 PerCP-Cy5.5 (53-6.7). Peanut agglutinin (PNA)-Cy5 (Galβ1,3GalNAc α1, O-Ser/Thr), *Sambucus nigra* (SN)-Cy5 (Neu5Ac(α2,6)Gal/GalNAc), and biotinylated *Maackia amurensis* II (MAL II, Neu5Ac(α2,3)Galβ3GalNAc) lectins were acquired from Vector Laboratories. *Amaranthus leucocarpus* lectin (ALL) recognizes GalNAc within Galβ1,3GalNAc α1, O-Ser/Thr or GalNAc α1, O-Ser/Thr structures, however unlike PNA, ALL can recognize its ligand despite the presence of sialic acid in the structure (30). This lectin was purified in-house as previously described (31) and biotinylated with the EZ-Link Sulfo-NHS-Biotin kit (Thermo Fisher) following the manufacturer’s instructions using a 1:2 lectin: biotin ratio. Biotinylated lectins were detected with Streptavidin-Brilliant Violet 421 (Strp-BV421) conjugate (Biolegend), and dead cells were excluded using Ghost Dye Red 780 (Tonbo Biosciences) or Zombie Green (Biolegend). Samples were analyzed in a MACSQuant Analyzer flow cytometer (Miltenyi Biotec) acquiring 150μl per sample.

### Cell staining

One million cells were first incubated with the lectin(s) from the corresponding panel (**Supplementary Table 2**), followed by a second incubation with Strp-BV421 when required and a final incubation with the indicated antibody mix. All incubations were performed in washing buffer (DPBS, 1% FCS) for 30 min at 4°C, in the darkness, and two washes were performed between each step. The final wash was carried out with DPBS; cells were then incubated with Ghost Dye Red 780 or Zombie Green in DPBS (20 min, RT), washed with washing buffer, and resuspended in 200 μl of DPBS. Samples were immediately analyzed by flow cytometry.

### Intracellular cytokine detection

Ten million splenocytes were incubated with eBioscience Cell Stimulation Cocktail plus protein transport inhibitors (Invitrogen, Thermo) in 2 ml complete RPMI medium (RPMI 1640 supplemented with 10% FCS, 2mM L-glutamine, 10 mM non-essential amino acids, 1mM sodium pyruvate, 25 mM HEPES, 50 µM 2-ME, and IU/ml penicillin-streptomycin [GIBCO]) in each well of a 24 well plate (Costar) for 6 h at 37° C in a humidified atmosphere containing 5% CO_2_ in air. Cells were harvested, washed, and stained with SN Cy5, the antibodies against surface molecules depicted in the **Supplementary Table 1** and Zombie Green as described above. Samples were resuspended in 100µl of 4% paraformaldehyde in DPBS, mixed gently for 10 min at RT for fixation, washed with Perm Buffer (DPBS, FCS 1%, saponin, 0.025%), and stained for intracellular cytokines for 10 min at RT, in darkness, using the same buffer. Finally, cells were washed in Perm Buffer, resuspended in 200 µl DPBS, and analyzed by flow cytometry.

### Data and statistical analysis

Flow cytometry data were analyzed using FlowJo Software V10.8 (Becton Dickinson) using the gating strategy depicted in **Supplementary Figures 1**–**4**, and statistical analysis was performed with the PRISM Software V9.2 (GraphPad). The Shapiro-Wilk test was used to assess data normal distribution and the Brown-Forsythe test for equal variances. Data with a normal distribution and equal variances were further analyzed using one-way ANOVA and Bonferroni’s multiple comparison test or two-tailed unpaired *t-*test; for data without a normal distribution, differences were determined using Kruskal-Wallis nonparametric test followed by Dunn’s multiple comparison test or two-tailed Mann-Whitney test. Finally, for data with a normal distribution but different variances, the Brown-Forsythe and Welch ANOVA tests followed by Dunnett T3 multiple comparison test or two-tailed unpaired *t*-test with Welch’s correction were used; in all cases, a minimum p< 0.05 value was considered statistically significant.

## Results

### Sial α2,6 downregulates in CD4^+^ and CD8^+^ splenocytes from mice acutely infected with *Toxoplasma gondii*

**Figure 1:**
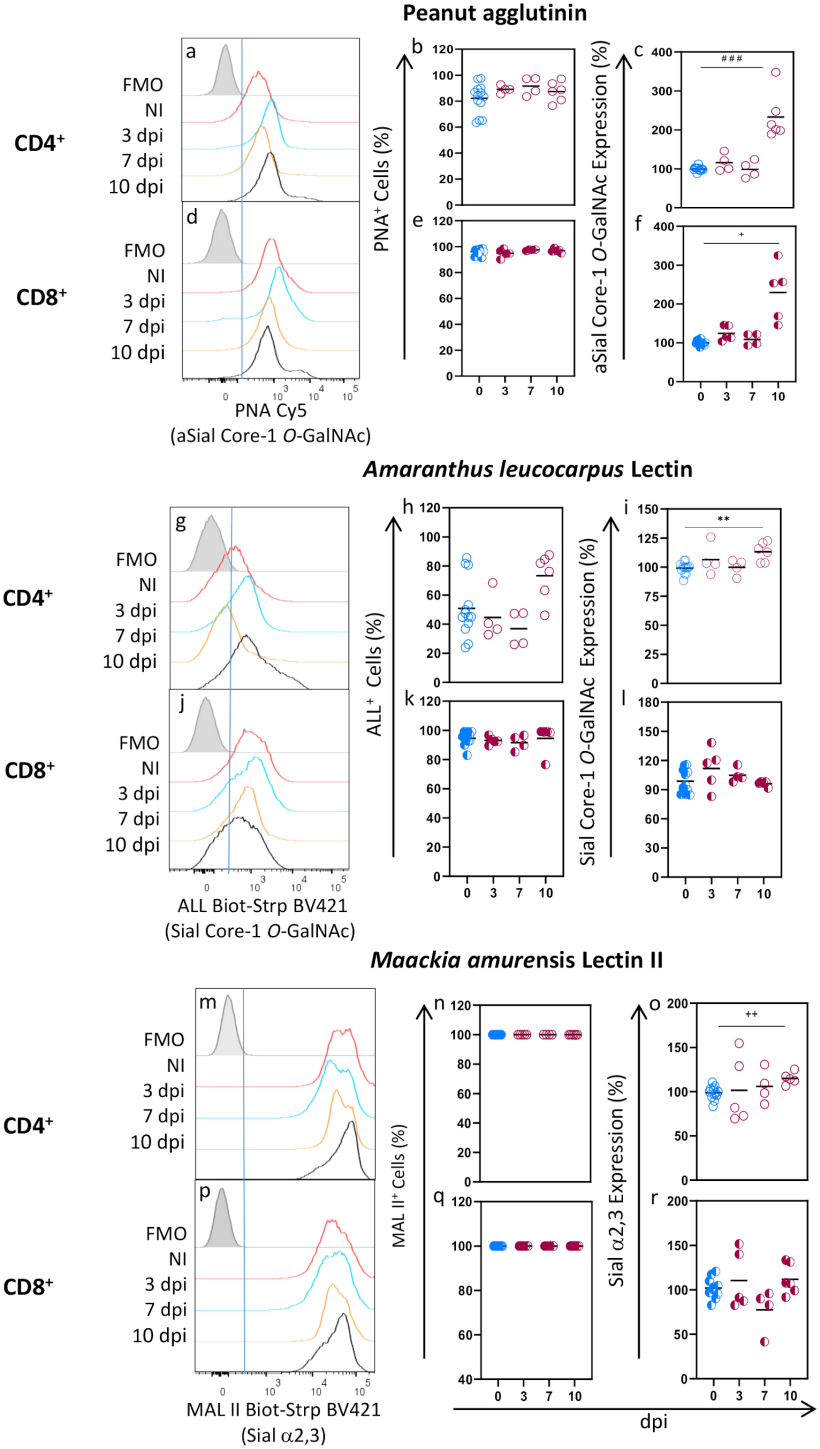
The proportion of CD4^+^ and CD8^+^ lymphocytes binding PNA, ALL, and MALII does not change during acute *T. gondii* infection. BALB/c Foxp3^EGFP^ mice were orally infected with 10 cysts from the ME49 strain, and spleens were obtained after 3, 7, and 10 days post^-^infection (dpi). Splenocytes were obtained, stained using the panels indicated in **Supplementary Table 1**, analyzed by flow cytometry, and gated as described in **Supplementary Figure 1**. Representative histograms are shown for PNA (a, d), ALL (g, j) and MALII (m, p) binding to CD4^+^ and CD8^+^ cells at 3 (blue line), 7 (yellow line), and 10 (black line) days post-infection, or from uninfected mice (red line); Fluorescence minus one (FMO) controls for all lectins are shown in gray. Data were obtained from 2 independent experiments for 3 and 7 dpi analysis and 3 independent experiments for 10 dpi analysis, including at least 2 mice per group and are expressed as % cells binding each lectin (b, e, h, k, n, q) and as a percentage of glycosylation expression (c, f, i, l, o, r) in CD4^+^ (○) and CD8+ (◐) cells. Geometric Mean Fluorescence intensity from infected animals (red) was normalized to the corresponding average value of uninfected mice (blue) per experiment and graphed as a percentage expression. Data were analyzed using one-way ANOVA with Bonferroni’s multiple comparison test, **p<0.001; Krustal-Wallis with Dunn’s multiple comparison test, ^# # #^<0.005; or the Brown-Forsythe and Welch ANOVA tests followed Dunnett T3 multiple comparison test, ^+^p<0.05, ^+ +^p<0.001.

We studied cell surface glycosylation patterns and changes in spleen CD4^+^ and CD8^+^ lymphocytes during acute *T. gondii* infection. To that end, we analyzed the binding of 4 lectins: Peanut agglutinin (PNA) and *Amaranthus leucocarpus* lectin (ALL), which bind asialylated and sialylated/asialylated core 1-*O*-GalNAc glycans, respectively, and *Maackia amurensis* lectin II (MAL II) and *Sambucus nigra* lectin (SN), which bind to α2,3 and α2,6 sialylated molecules (Sial α2,6 and Sial α2,3), respectively (**Supplementary Table 2**). The binding patterns and changes of these lectins on conventional (Foxp3^-^) CD4^+^ and CD8^+^ lymphocytes were studied by flow cytometry using the strategy depicted in **Supplementary Figure 1**. We found that PNA lectin bound 82.19% of CD4^+^ and 96.15% of CD8^+^ lymphocytes from uninfected mice (**Figures 1a, b, d, e**) indicating that most cells of these subtypes express asialylated l-Core1 *O*-GalNAc glycans on the surface. Although the percentage of PNA^+^ cells, either CD4^+^ or CD8^+^, did not significantly change during *T. gondii* infection (**Figures 1b, e**), PNA expression did increase as infection progressed, and at 10 dpi a significant 2- and 1.5-fold increase was observed in CD4^+^ and CD8^+^ cells (**Figures 1c, f**), respectively. These results indicate that aSial Core-1 *O-*GalNAc glycan expression increased in both cell subtypes during infection.

In agreement with previous reports (32, 33), ALL binding was different between CD4^+^ and CD8^+^ cells from uninfected mice since only 50.85% of CD4^+^ cells and most CD8^+^ cells (94.54%) were ALL^+^ (**Figures 1g, h, j, k**). CD4^+^ ALL^+^ cells increased at 10 dpi, but it was not statistically significant (**Figure 1h**); the percentage of CD8^+^ ALL^+^ cells, however, remained unchanged during infection (**Figure 1k**) while a slight increase (13%) in CD4^+^ ALL^+^ cells was observed only at 10 dpi (**Figure 1i**). Accordingly, Sial core-1 *O-*GalNAc glycans expression remained unchanged through infection in CD8^+^ cells (**Figure 1l**) and slightly increased at 10 dpi in CD4^+^ cells only (**Figure 1i**). In contrast, all CD4^+^ and CD8^+^ cells expressed the Sial α2,3 residue recognized by MAL II lectin (**Figures 1m, n, p, q**). We found that this glycosylation was highly expressed by both cell subtypes, as shown by a very high MFI, which remained unchanged during infection (**Figures 1m, pm, o, r**).

**Figure 2:**
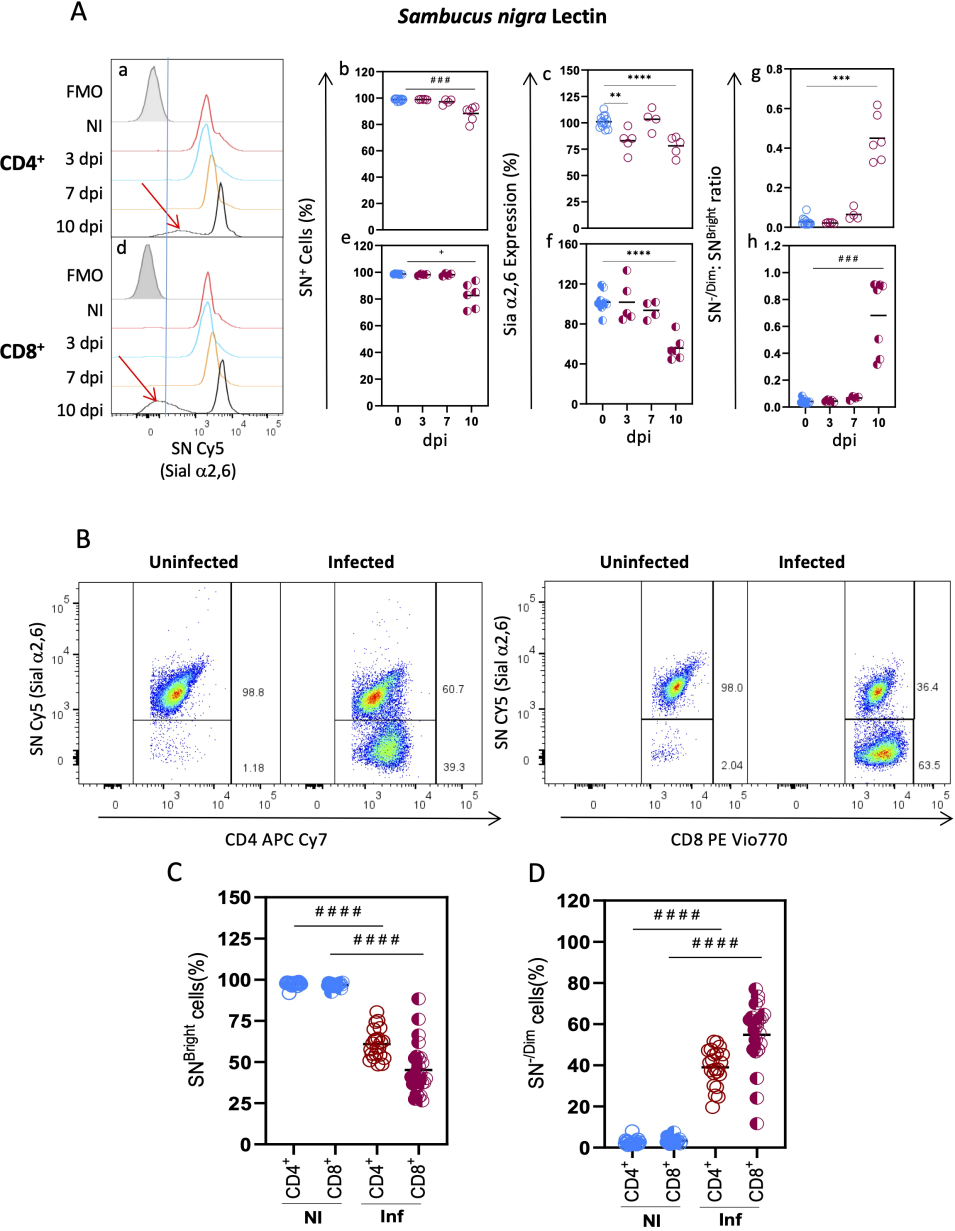
Decreased Sial α2,6 expression is a CD4^+^ and CD8^+^ hallmark event after *T. gondii* infection. Mouse splenocytes obtained at different dpi, as depicted in **Figure 1**, were stained using the panels indicated in **Supplementary Table 1**, analyzed by flow cytometry, and gated as described in **Supplementary Figure 1**. **(A)** Representative histograms are shown for SN binding to CD4^+^ (a) and CD8^+^ (d) cells at 3 (blue line), 7 (yellow line), and 10 (black line) dpi, or from uninfected mice (red line); FMO controls are shown in gray. Red arrows highlight the SN^-/Dim^ populations found at 10 dpi (a, d). CD4^+^ (○) and CD8^+^ (◐) cells, data were obtained from 2 independent experiments for 3 and 7 dpi analysis and 3 independent experiments for 10 dpi analysis, including at least 2 mice per group, and are expressed as percentage SN^+^ binding cells (b, e), levels of Sial α2,6 expression (c, f), and the [% SN^-/Dim^ cells]: [% SN^Bright^ cells] ratio (g, h). In (c, f), geometric mean fluorescence intensity from infected animals (red) was normalized to the corresponding average value of uninfected mice (blue) per experiment and graphed as a percentage expression. **(B)** Representative dot plots are shown for SN binding within CD4^+^ and CD8^+^ cells at 10 dpi and from uninfected mice. The percentage of SN^Bright^ **(C)** and SN^-/Dim^ **(D)** cells from uninfected (NI, blue) and infected (Inf, red) mice within CD4^+^ (○) and CD8^+^ (◐) lymphocytes is depicted; data were obtained from 7 independent experiments, including at least 2 mice per group. Statistical analysis was performed using one-way ANOVA with Bonferroni’s multiple comparison test, **p<0.001, ***p<0.005, ****p<0.0001; Krustal-Wallis with Dunn’s multiple comparison test, ^# # #^p<0.005, ^# # # #^p<0.0001 or the Brown-Forsythe and Welch ANOVA tests followed Dunnett T3 multiple comparison test, ^+^p<0.05.

SN lectin binding was similar to MAL II on both subtypes since most (>98%) CD4^+^ and CD8^+^ cells from uninfected animals were SN^+^ and expressed an elevated density of Sial α2,6 linkage surface glycoconjugates (**Figures 2A-a, b, d, e**). No changes were detected at early times after infection (3 and 7 dpi). However, at 10 dpi, a slight reduction of CD4^+^ SN^+^ and CD8^+^SN^+^ cells (10.58 and 16.33%, respectively) was observed (**Figures 2A-b, e**) along with a statistically significant decrease of their Sial α2,6 expression (**Figures 2A-c, f**). Moreover, as can be seen in the SN binding histograms (**Figures 2A-a, d**. red arrows), an SN^-/Dim^ cell population emerged at 10 dpi in both subtypes, indicating the loss of Sial α2,6 as a consequence of infection. The [SN^Bright^ cells: SN^-/Dim^ cells] ratio at each time point showed that the SN^-/Dim^ cells started to expand at 7 dpi (**Figures 2A-g, h**). Overall, these results show that each of the tested lectins bound CD4^+^ and CD8^+^ cells with different patterns, both among lectins and between subtypes. Although CD4^+^ and CD8^+^ glycophenotypes are different and are modified during acute *T. gondii* infection, SN was the only lectin whose binding revealed a novel T cell population that lost Sial α2,6 due to infection.

The unexpected appearance of such a distinctive population (SN^-/Dim^ cells) arising at 10 dpi prompted us to further examine it at this particular time point. We analyzed the proportion of CD4^+^ and CD8^+^ cells that were SN^Bright^ and SN^-/Dim^ and found that most cells (either CD4^+^ or CD8^+^) were SN^Bright^ (>96%) in uninfected mice (**Figures 2B, C**). At 10 dpi, a reduction of 30% and 50% of SN^Bright^ CD4^+^ and CD8^+^ cells, respectively, was detected (**Figure 2C**); consequently, a high proportion (40-60%) of both subtypes were essentially SN^-/Dim^ cells at this time point (**Figure 2D**). Sial α2,6 expression in the remaining SN^Bright^ cells remained unchanged (data not shown). These results demonstrate that losing Sial α2,6 expression is a hallmark event for T cells after infection, which is probably related to activation.

### Reduced Sial α2,6 expression correlates to activation progression and IFN-γ production in CD4^+^ and CD8^+^ cells

**Figure 3:**
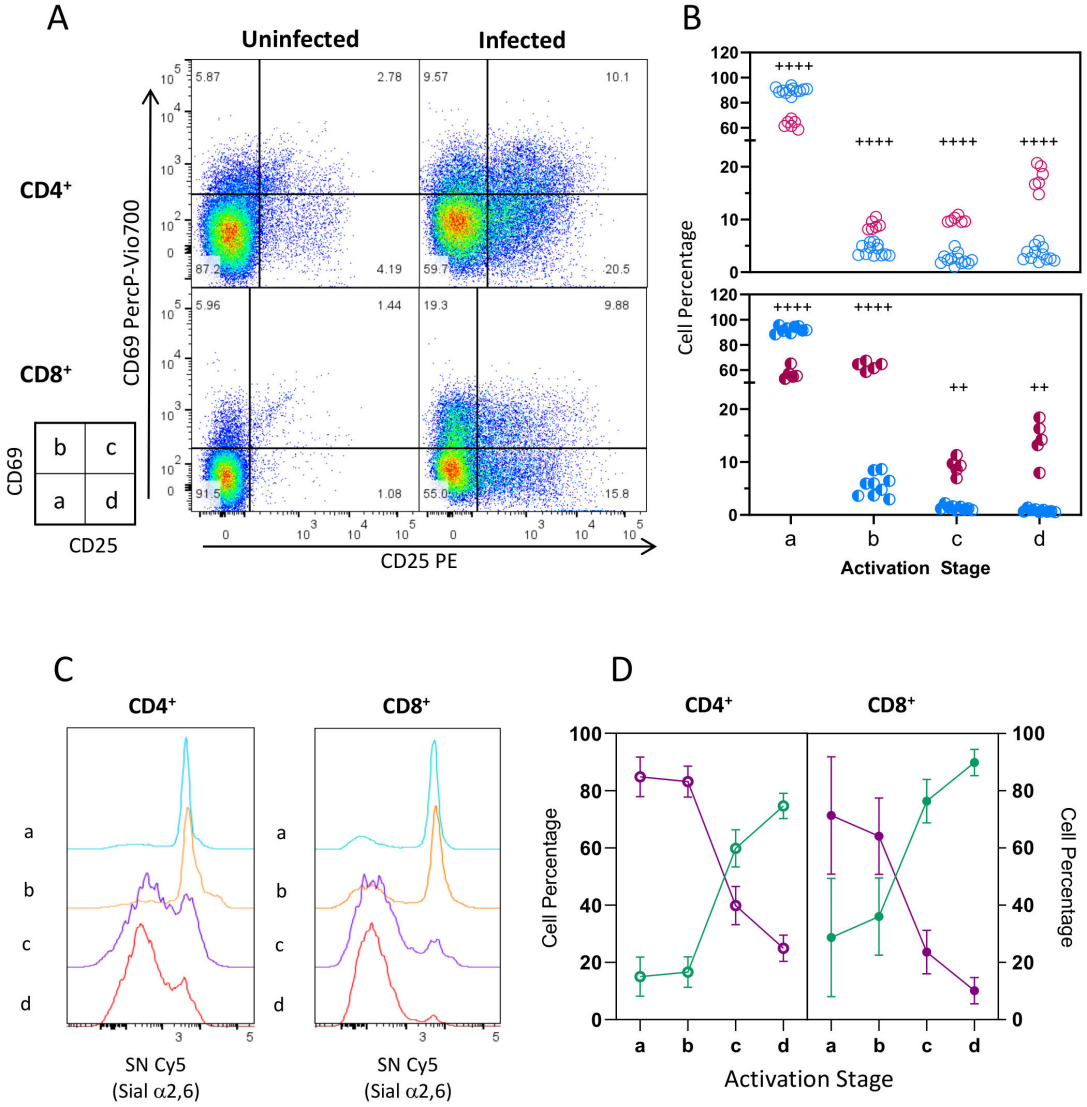
Sial α2,6 expression decreases in CD4^+^ and CD8^+^ lymphocytes as activation progresses. Mouse splenocytes obtained at 10 dpi, as depicted in **Figure 1**, were stained using the panels indicated in **Supplementary Table 1**, analyzed by flow cytometry, and gated as described in **Supplementary Figure 2**. **(A)** Representative dot plots show CD69 and CD25 expression in CD4^+^ and CD8^+^ cells from uninfected animals or at 10 dpi. Four activation stages were identified: Stage *a* (CD69^-^ CD25^-^, resting cells), Stage *b* (CD69^+^ CD25^-^, early activated cells), Stage *c* (CD69^+^ CD25^+^, intermediate activated cells), and Stage *d* (CD69^-^ CD25^+^, late activated cells). **(B)** CD4^+^ (○) and CD8^+^ (◐) cell percentage per stage from infected (INF, red) and uninfected (NI, blue) animals. **(C)** Representative histograms showing SN binding on CD4^+^ and CD8^+^ cell subtypes on each activation stage at 10dpi. **(D)** Percentage of SN^Bright^ (purple) and SN^-/Dim^ (green) cells at 10 dpi. Data were obtained from 3 independent experiments for 10 dpi analysis, including at least 3 mice per group, and analyzed using the Brown-Forsythe and Welch ANOVA tests followed by Dunnett T3 multiple comparison test, ^+ +^<0.001, ^+ + + +^ p<0.0001.

We thus determined if Sial α2,6 downregulation detected by SN binding during infection was related to activation progression. To that end, we used CD69 and CD25 expression to identify cells at different activation time points (**Figure 3A**): resting cells (*stage a*: CD69^-^ CD25^-^), early (*stage b*: CD69^+^ CD25^-^), intermediate (*stage c*: CD69^+^ CD25^+^), and late activated cells (*stage d*: CD69^-^ CD25^+^). As expected, most CD4^+^ and CD8^+^ cells from uninfected mice were on *stage a* (**Figures 3A, B**. 89.87% and 92.22%, respectively), and very few cells were on stage *b-d* (<10%). At 10 dpi, a sizable proportion of activated lymphocytes (stage b-d) was apparent in both cell subtypes (**Figures 3A, B**). In contrast, a lower percentage of cells on *stage a* were detected (~60%), indicating that cells were indeed activated due to the infection. Notably, CD4^+^ cells activate faster than CD8^+^ since most activated CD4^+^ cells express CD25 only (*stage d*, 17.98%), while most CD8^+^ cells are primarily early activated CD69^+^ CD25^-^ cells (*stage b*, 63.36%).

Next, we analyzed SN binding in the stages mentioned above. Representative histograms (**Figure 3C**) indicated that CD4^+^ and CD8^+^ cell subtypes are SN^Bright^ during the first 2 activation steps but lost SN binding as activation progressed to *stages c* and *d*. Data from different experiments (**Figure 3D**) showed that most CD4^+^ (64.1%) and CD8^+^ (84.8%) cells in *stages a* and *b* are SN^Bright^ and only a small proportion (15% and 25%, respectively) are SN^-/Dim^; however, 60-80% of cells in *stage c* are SN^-/Dim^, this proportion increased on *stage d*, turning the SN^-/Dim^ population into the predominant one. The increasing SN^-/Dim^ cell population observed through the defined stages during infection indicates that Sial α2,6 downregulation is related to CD4^+^ and CD8^+^ activation progress.

**Figure 4:**
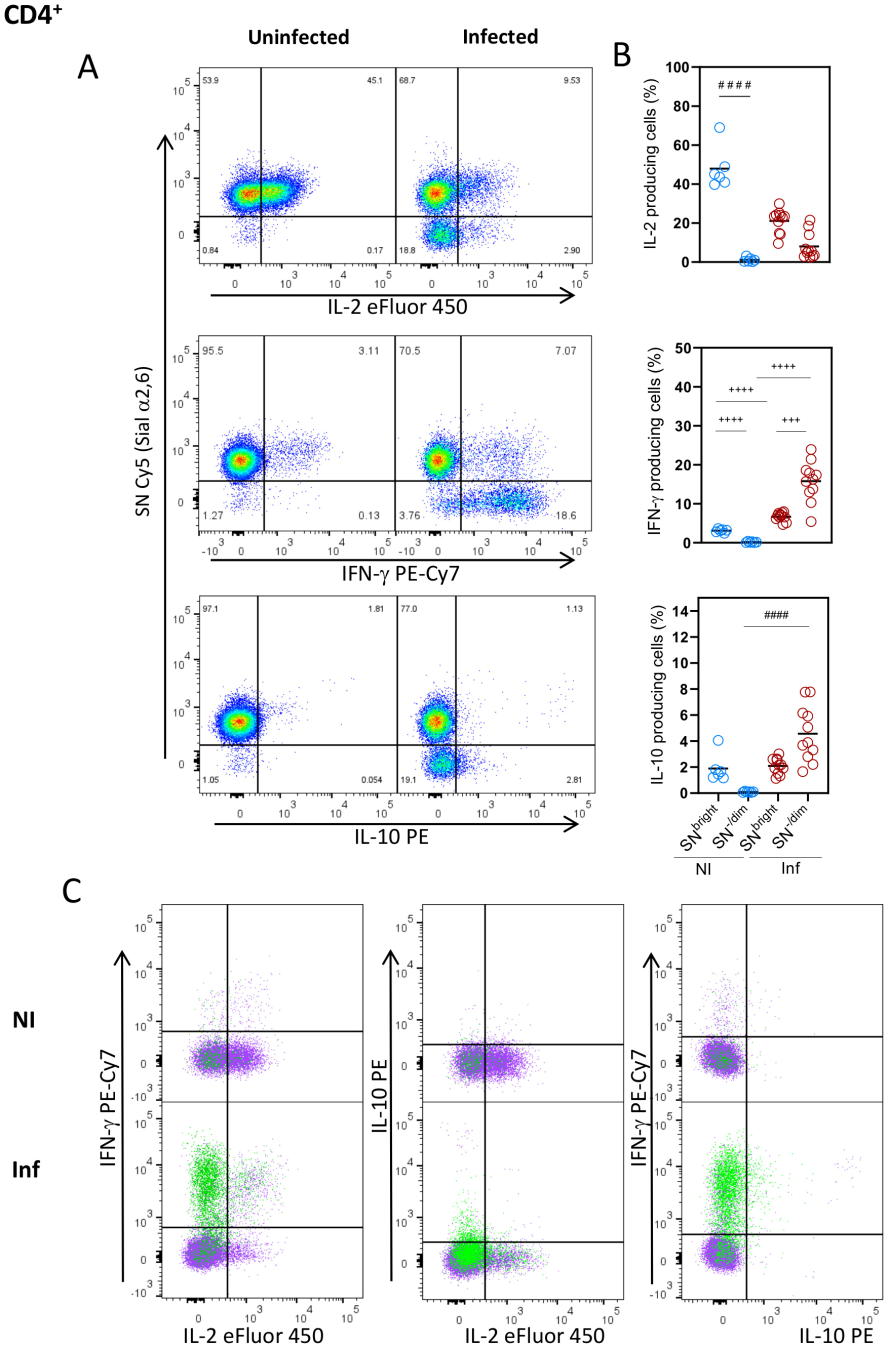
Sial α2,6 expression level relates to cytokine production patterns in CD4^+^ lymphocytes. Mouse splenocytes obtained at 10 dpi were stained using the panels indicated in **Supplementary Table 1**, analyzed by flow cytometry, and gated as described in **Supplementary Figure 3**. **(A)** Representative dot plots showing IL-2, IFN-γ and IL-10 produced by CD4^+^ cells from uninfected (NI) or infected (INF) mice. **(B)** Statistical analyses showing the percentage of IL-2, IFN-γ and IL-10 production in SN^Bright^ and SN^-/Dim^ CD4^+^ cells (○) from NI (blue) and INF (red) mice. **(C)** Overlaid data from SN^Bright^ (purple) and SN^-/Dim^ (green) is depicted. Cells were obtained from 3 independent experiments, including at least 2 mice per group. Data were analyzed using the Krustal-Wallis with Dunn’s multiple comparison test, ^####^ p<0.0001, or the Brown-Forsythe and Welch ANOVA tests followed by Dunnett T3 multiple comparison test, ^+ + +^<0.0005, ^+ + + +^ p<0.0001.

**Figure 5:**
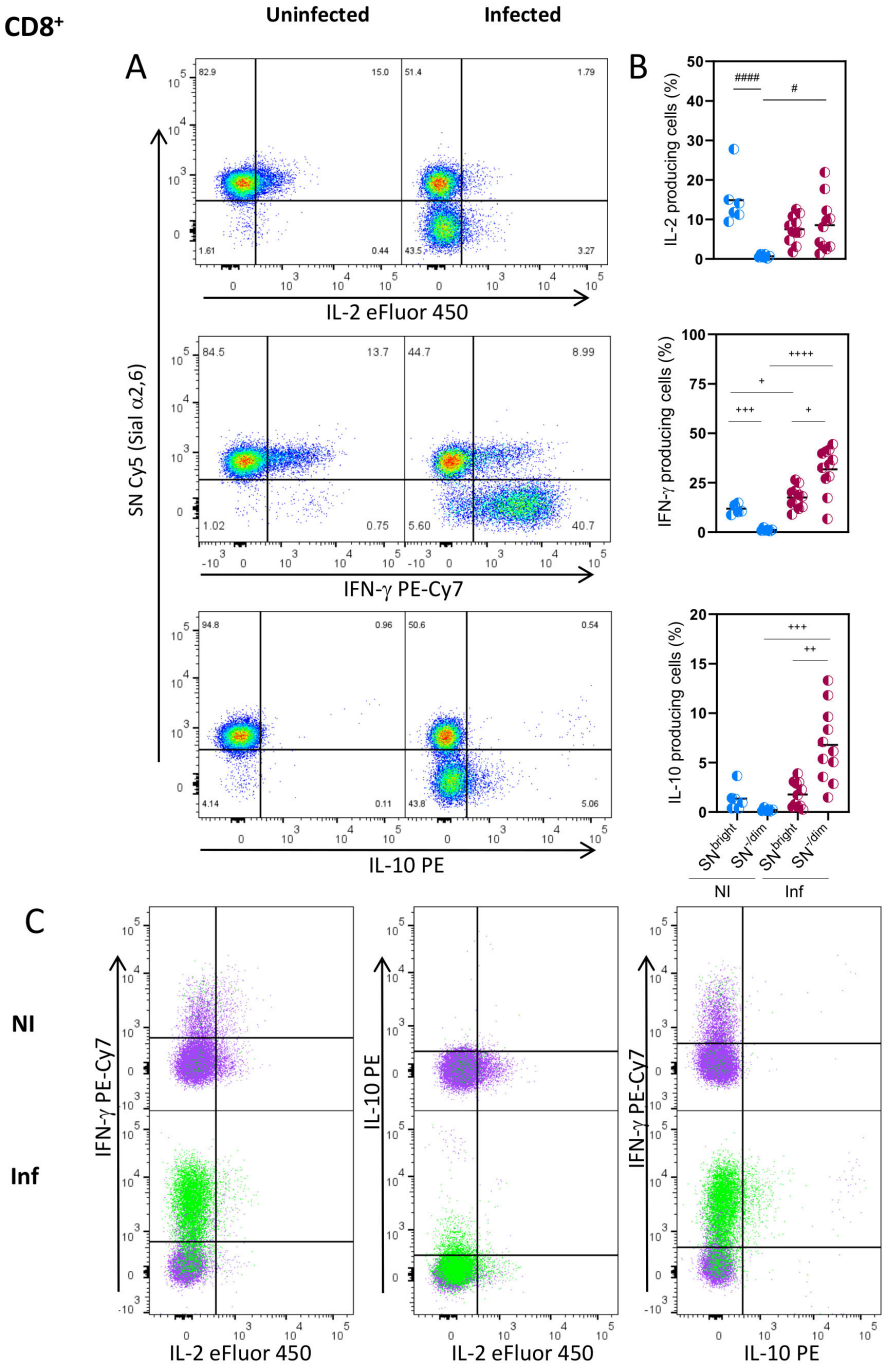
Sial α2,6 expression level relates to cytokine production patterns in CD8^+^ lymphocytes. Mouse splenocytes obtained at 10 dpi were stained using the panels indicated in **Supplementary Table 1**, analyzed by flow cytometry, and gated as described in **Supplementary Figure 3**. **(A)** Representative dot plots showing IL-2, IFN-γ and IL-10 produced by CD8^+^ cells from uninfected (NI) or infected (INF) mice. **(B)** Statistical analyses showing the percentage of IL-2, IFN-γ and IL-10 production in SN^Bright^ and SN^-/Dim^ CD8^+^ cells (◐)from NI (blue) and INF (red) mice. **(C)** Overlaid data from SN^Bright^ (purple) and SN^-/Dim^ (green) is depicted. Cells were obtained from 3 independent experiments, including at least 2 mice per group. Data were analyzed using the Krustal-Wallis with Dunn’s multiple comparison test, # p<0.05, #### p<0.0001, or the Brown-Forsythe and Welch ANOVA tests followed by Dunnett T3 multiple comparison test, ^+^p<0.05, ^+ +^p<0.001 ^+ + +^<0.0005, ^+ + + +^ p<0.0001.

To further understand the relationship between Sial α2,6 expression and T cell functionality, we analyzed the cytokines produced by the SN^-/Dim^ and SN^Bright^ cells. As observed in **Figures 4A, B**, CD4^+^ cells from uninfected mice produced mainly IL-2, all of which had the SN^Bright^ glycophenotype, while very few cells producing IFN-γ or IL-10 were detected. As expected, in *T. gondii* infected mice, the main cytokine produced was IFN-γ (4, 34), whose detection along with IL-10 was, however, was mainly produced by the SN^-/Dim^ population. Both glycophenotypes produced IL-2, although to a lesser extent than non-infected animals, showing reduced IL-2 production in *T. gondii* infected mice; this observation agrees with previous reports (35). To further verify that each glycophenotype produced independent cytokine patterns, we analyzed the simultaneous production by both subtypes. **Figure 4C** shows that after infection, most IFN-γ and IL-2 are produced by independent populations, the SN^-/Dim^ and SN^Bright^ cells correspondingly. A small SN^-/Dim^ population produces both IFN-γ and IL-2, and a small amount of IL-10 was simultaneously detected with IFN-γ by SN^-/Dim^ cells. A similar cytokine production pattern was observed on CD8^+^ cells (**Figures 5A–C**), that is, IFN-γ producing cells showed the SN^-/Dim^ phenotype and IL-2 producing cells showed mainly an SN^Bright^ phenotype in infected animals; a small number of IL-10 producing cells was detected showing a SN^-/Dim^ phenotype. These results show that T_H1_ and T_C1_ polarized cells can be further sub-classified according to the predominant cytokine produced, and this is directly related to Sial α2,6 expression.

### Sial α 2,6 expression distinguishes a new active state in CD4^+^ and CD8^+^ cells

**Figure 6:**
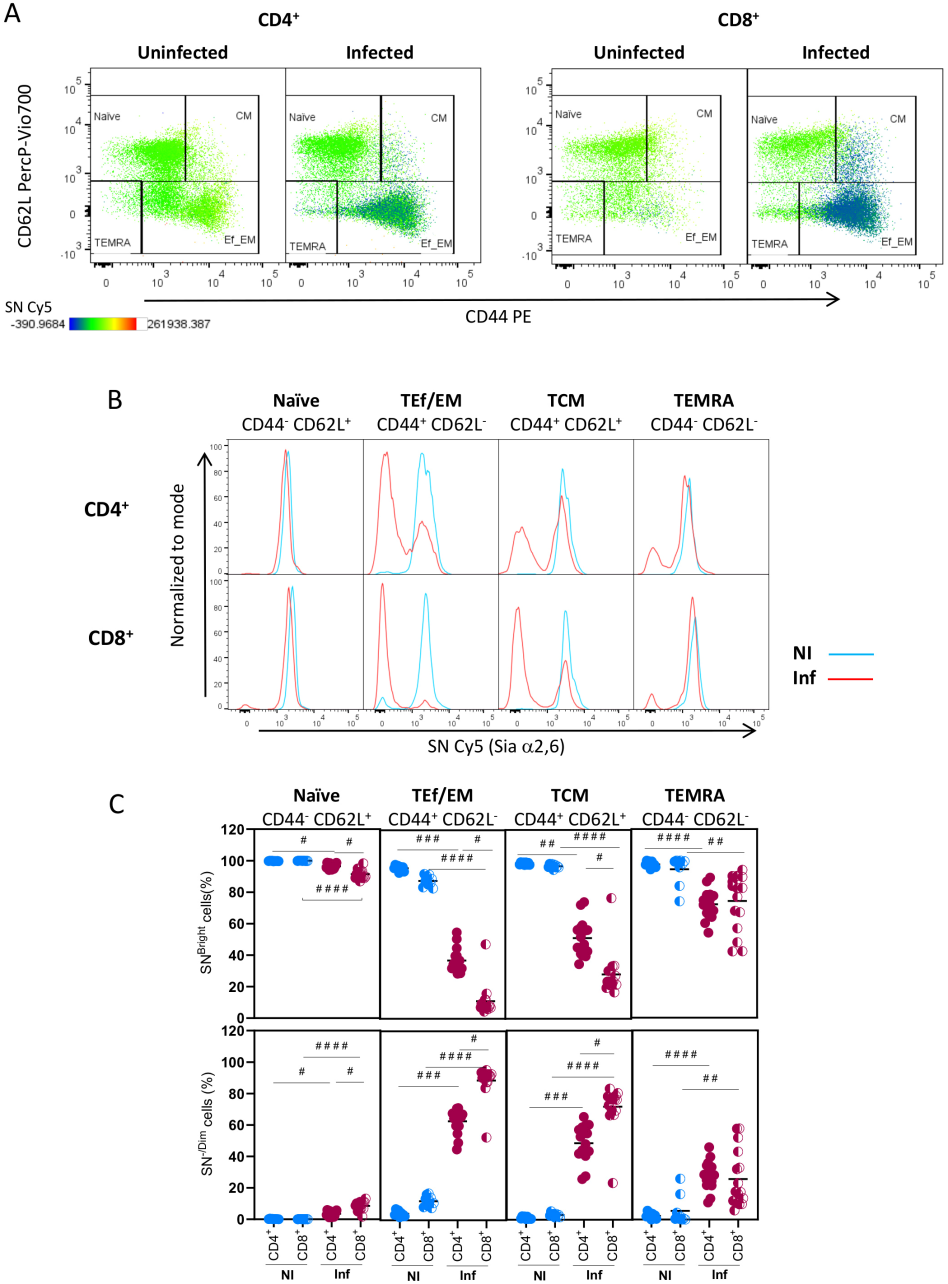
TEf/TCM and TCM subpopulations from CD4^+^ and CD8^+^ cells generated during acute *T. gondii* infection are mainly Sial α2,6^-/Dim^. Mouse splenocytes obtained at 10 dpi were stained using the panels indicated in **Supplementary Table 1**, analyzed by flow cytometry, and gated as described in **Supplementary Figure 4**. **(A)** Representative dot plots for CD44, CD62L, and SN (Sial α2,6) analysis in CD4^+^ and CD8^+^ cells are shown. **(B)** Representative histograms of SN expression in naïve (CD62L^+^ CD44^-/Dim^), effector/effector memory T cells (TEf/EM, CD62L^-^CD44^Higth^), central memory cells (CM, CD62L^+^ CD44 ^Higtht^), and terminally differentiated effector memory cells (TEMRA, CD62L^-^ CD44^-^) within CD4^+^ and CD8^+^ cells from uninfected (NI, blue) and 10 dpi infected animals (Inf, red). **(C)** Percentage of SN^Bright^ and SN^-/Dim^ cells within CD4^+^ (○) or CD8^+^ (◐) naïve, TEf/EM, TCM, and TEMRA subpopulations from uninfected (NI, blue circles) and 10 dpi infected animals (Inf, red circles). Data were obtained from 3 independent experiments, including at least 3 mice per group. Data were analyzed using Krustal-Wallis with Dunn’s multiple comparison test, ^#^p<0.05, ^# #^p<0.001, ^# # #^p<0.005, ^# # # #^p<0.0001.

**Figure 7:**
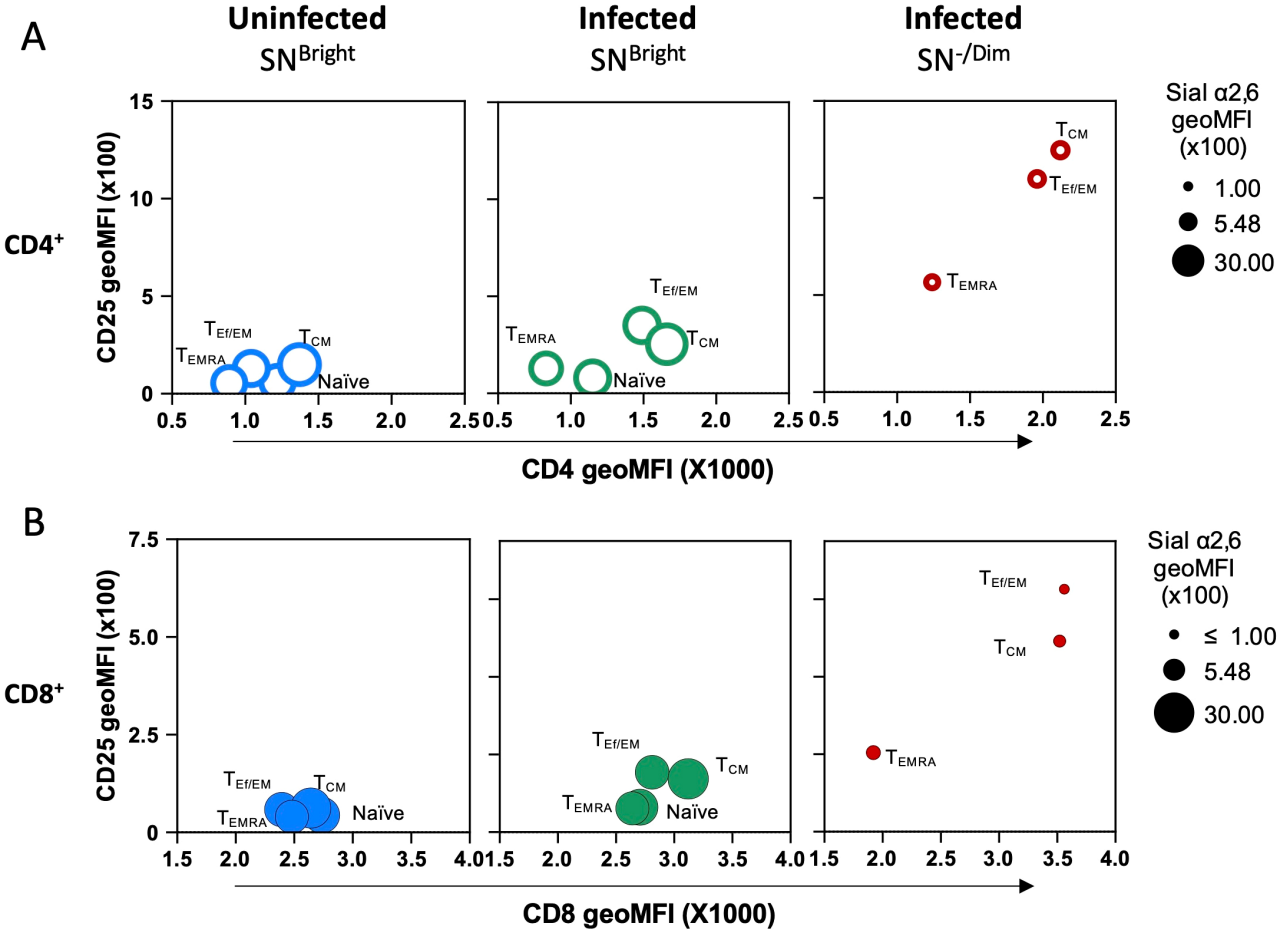
Sial α2,6 expression distinguishes quiescent and infection-induced active CD4^+^ and CD8^+^ lymphocytes during acute *T. gondii* infection. Average CD25, CD4, or CD8 and Sial α2,6 geoMFI from SN^Bright^ (quiescent) and SN^-/Dim^ (active) naïve, TEf/EM, TCM, and TEMRA subpopulations defined in **Figure 7** were compared within CD4^+^ (**A**, ○) and CD8^+^ (**B**, ●) subsets using bubble plots. Phenotypic characteristics are observed comparing **(A)** CD4 or **(B)** CD8 expression (x-axis), CD25 expression (y-axis), and Sial α2,6 expression (circle perimeter). Raw data and statistical analysis are found in **Supplementary Figure 5**.

Then, we analyzed how Sial α2,6 expression relates to T cell differentiation. To this end, we determined SN binding on each T-cell subpopulation identified by CD62L and CD44 expression: naïve (CD62L^Bright^ CD44^-/Low^), central memory (CM) (CD62L^High^ CD44^High^), effector or effector memory (Ef/EM) (CD62L^-/Low^ CD44^High^), and TEMRA (CD62L^-^ CD44^Low/Int^) cells (27, 28). These subpopulations were clearly detected in CD4^+^ and CD8^+^ subtypes from uninfected and *T. gondii* infected mice (**Figure 6A**). Results showed that in both CD4^+^ and CD8^+^ naïve cells, either from uninfected or from infected mice, accounted for >91% of SN^Bright^ cells and expressed similar high amounts of Sial α2,6 (**Figures 6A–C**). TEf/EM and TCM from uninfected mice all show high SN binding, but in infected mice, a high proportion of cells became SN^-/Dim^, mostly in CD8^+^ cells; a similar pattern was observed in T_CM_ cells (**Figures 6B, C**). In TEMRA cells, the emergence of SN^-/Dim^ cells was more discreet (≤ 40%) in infected mice for both CD4^+^ and CD8^+^ cells. Since the SN^-/Dim^ population is detected only in infected animals, these results strongly suggest that Sial α2,6 allows the differentiation of quiescent cells (SN^Bright^) from infection-induced active cells (SN^-/Dim^). A final analysis (**Figure 7**) demonstrates that all differentiated subpopulations of active cells (SN^-/Dim^) exhibit a distinctive pattern with high CD25 and CD4/CD8 expression levels. In contrast, quiescent cells (SN^Bright^), either from uninfected or infected animals, show a lower CD4/CD8 and CD25 expression pattern. This confirms that in the absence or presence of infection, quiescent cells present similar characteristics that clearly differentiate them from infection-induced active cells and that they can be differentiated through their Sial α2,6 expression.

## Discussion

This work aimed to analyze sialylation dynamics in conventional CD4^+^ and CD8^+^ subtypes during *T. gondii* infection. We wanted to analyze previously reported glycosylations involved in T cell activation and/or maturation, like Sial α2,3, Sial α2,6 linkages, and asialylated/sialylated Core-1 *O*-GalNAc glycans. To this end, we first evaluated the binding of SN, MAL II, PNA, and ALL lectins by flow cytometry in *T. gondii* infected mice splenocytes during the first ten days of infection.

Analyses revealed that while the percentage of CD4^+^ and CD8^+^ cells binding PNA, ALL, and MAL II remained constant through infection, a slight expression increase in the glycans recognized by these lectins was found at ten dpi, particularly within the CD4^+^ population. PNA recognizes the OH-in C4 of Gal in the T-antigen, and ALL recognizes the OH- on C4 and the C-2 acetamido groups of the reduced GalNAc residue of the T-antigenic disaccharide; the former, but not the latter interaction is blocked by the presence of sialic acid at C-3 or C6 (30). Glycans recognized by these two lectins are essential during T cell ontogeny since their differential expression relates to T cell maturation and thymus location. PNA binds mainly to cells found at the thymus cortex and ALL to cells at the medulla, which correlates with immature and mature lymphocytes, respectively (18, 36, 37). Loss of the PNA^+^ glycophenotype is related to an increased α2,3 sialyltransferase expression (38), which could explain the PNA-ALL^+^ mature T cell glycophenotype observed in the thymus. Our results show that all mature CD4^+^ and CD8^+^ cells are PNA^+^ and that PNA ligands expression increases at ten dpi. These agree with the previously reported molecules recognized by this lectin (17, 23, 39); CD8, CD43, and CD45, among which the latter has been shown to convey most PNA ligands in activated CD8^+^ lymphocytes (40). Contrastingly, we found that while all CD8^+^ are ALL^+^, half of CD4^+^ cells bind ALL, and only a slight expression increase was observed at 10 dpi in the CD4^+^ subtype. Our observations agree with previous reports showing the same glycophenotype in CD4^+^ and CD8^+^ cell subtypes from healthy mouse splenocytes (32, 33). Additionally, the primary ALL ligand, Moesin, can provide a costimulatory signal, equivalent to CD28, *in vitro* that induces T cell activation, proliferation, and IL-2 production, suggesting that ALL ligands could be more related to signal amplification or maintenance at very early activation points rather than being involved in differentiation (33, 41). MAL II recognizes the Sial α2,3 linkage, critical for cell interactions, as described for human malignancies, T and B cell communication, and bacterial and viral infection (42–45). Although this moiety was found at the highest cellular density in all conventional CD4^+^ and CD8^+^ cells, only a subtle statistically significant increase was observed at ten dpi in the former population. The same analyses were performed on T_reg_ (Foxp3^+^) cells and no differences were found (data not shown), which agrees with previous reports (46). Interestingly, it has been described that *T. gondii* PLK tachyzoites attach preferentially to cells expressing higher levels of Sial α2,3 than Sial α2,6 *in vitro* (45); the ubiquitous Sial α2,3 cell expression could partly explain *T. gondii* ability to infect a wide variety of cell types.

SN recognizes Sial α2,6 linkage within the minimal determinant Neu5Acα2-6Galβ1-4GlcNAc (47); it is crucial for leukocyte adhesion, neutrophil transmigration (48, 49), fertility (50), and cell interaction between cancerous cells (51). SN binding analysis showed that Sial α2,6 expression in CD4^+^ and CD8^+^ cells from uninfected animals is constitutive and high. However, at 10 dpi, we found a distinctive population with an SN^-/Dim^ glycophenotype in both cell subtypes. A similar observation has been reported in human CD4^+^ cells, where after *in vitro* polyclonal activation, Sial α2,6 downregulates under T_H1_ polarizing conditions (25). However, this is the first time that such glycophenotype is described using a T_H1_ polarizing infectious model, demonstrating that Sial α2,6 downregulation is not limited to the CD4^+^ subtype and occurs *in vivo* due to an immunological response.

Infection with *T. gondii* induces a very strong T_H1_ immune response characterized by the production of high levels of IL-2 and IFN-γ, but also of TNF-α, IL-1β, IL-10, IL-27, and IL-13 (1). Innate immune response is essential for initiating the protective immune response against *T. gondii*. TLR11 and TLR12 expressed by CD8^+^ dendritic cells recognize a profilin-like protein (TgPRF) produced by the parasite (52, 53), and upon recognition of this protein, dendritic cells produce IL-12; this cytokine, in turn, stimulates the production of high levels of IFN-γ by NK cells and CD4^+^ T cells, leading to protection of the host (54). Thus, the parasite itself is responsible for the induction of the strong T_H1_ response. It is tempting to speculate that such a polarized T_H1_ immune response is responsible for the downregulation of Sial α2,6 expression observed in CD4^+^ and CD8^+^ cells of infected mice, since Toscano et al. demonstrated *in vitro* that activated CD4^+^ T cells under T_H1_ and T_H17_, but not under T_H2_ conditions, showed downregulation of Sial α2,6 expression (25). Analysis of a different infection inducing a T_H1_ response would demonstrate if this hypothesis is true. Whether molecules produced by the parasite are also responsible for such downregulation remains to be established.

Previous reports describe that Sial α2,6 downregulation provides a signal that increases apoptotic sensitivity (25), susceptibility to galectin-1-induced cell death (25), and phagocytosis of apoptotic lymphocytes (55). Viability analysis of our data showed that in both uninfected and infected animals, dead cells are primarily Sial α2,6, especially within the CD8^+^ cell subset (**Supplementary Figure 6**). Thus, it is tempting to speculate that decreased Sial α2,6 expression in IFN-γ−producing CD4^+^ and CD8^+^ subsets described herein also works as a regulating signal that helps to contract the response against the parasite. Future experiments analyzing these cells at longer time points and anatomic sites after infection would provide more insight into this idea.

Further characterization of CD4^+^ and CD8^+^ SN^-/Dim^ populations showed that these are highly activated cells, as depicted by CD69 and CD25 expression, with a TEf/EM or TCM phenotype, as defined by their CD44 and CD62L expression pattern that produce high IFN-γ levels. This agrees with previous reports demonstrating that in T cells, particular glycosylations relate to specific cytokine production patterns. For example, cells with reduced *N*-glycosylation of TCR chains secrete higher levels of IL-2 and IFN-γ (56) and CD4^+^ T cells expressing β1,6GlcNAc-branched *N*-glycans produce higher IL-4 and lower IFN-γ, promoting a T_H2_ microenvironment (57).

Recently, transcriptomic analyses have provided further insight into T cell heterogeneity and function, opening a perspective that now includes timing and novel transitional subsets (12, 58). Such complexity keeps expanding as more detailed analyses are performed; in our case, determining sialic acid expression adds another layer to the already complicated protein analysis. This is moving us to redefine the vocabulary we use to describe T cells. For example, Cano et al. coined the term *effectorness*, referring to the CD4^+^ T cell potential to initiate a rapid and robust response upon stimulation (58). Chung et al. have discussed the necessity of talking about cell types, fates, and states by considering their stability, function, and temporality rather than limiting our description to subsets as defined by phenotype (13).

Traditionally, the terms subsets and subpopulations are used indistinctively in T cell analysis regardless of the combination of molecules used for phenotyping. Herein, we phenotyped cells regarding lineage, activation, differentiation, and glycosylation. Although each phenotypic analysis showed clear results (**Figures 2**–**7**), we still believe that a richer cell hierarchy depiction and more descriptive and integrative terms are needed to describe T cells better. To this end, we unified the terms used in this work using “*subtype*” to indicate lineage, “*subset*” to describe polarization, “*subpopulation*” for differentiation, and “*stage*” to describe steps within a process. Furthermore, we propose the use of the term “*state*” to describe the populations that we characterized herein; this is, we consider that SN^Bright^ cells are in a “quiescent state” and SN^-/Dim^ cells in an “active state”. Thus, we use the term “*state*” as a result of the global analysis of different phenotypes.

Our results reveal Sial α2,6 glycophenotyping as a novel, robust immune response analysis tool to easily detect active cells since it can be distinguished with a simple and single *ex vivo* staining without secondary *in vitro* activation processes to induce proliferation or activation marker expression (59). Moreover, this observation is not restricted to BALB/c mice, since the same results were obtained using mice from the C57BL/6J strain. Purifying the SN^-/Dim^ state generated after infection could allow the identification of the complete TCR antigen repertoire of infection–specific expanded clones, which can be a determinant for the generation of more effective vaccines. *In vitro* experiments have shown that downregulation of Sial α2,6 observed after activation occurs only under T_H1_ and T_H17_ but not T_H2_ conditions (25); thus, it remains to be determined if similar behavior is observed using other *in vivo* models with equivalent cell polarizations. It could also be an effective tool to study T cell proliferation and differentiation biology. Previous studies in CD8^+^ T cells show that after *in vivo* activation, a single naïve cell differentiates into both effector and memory cells (60), a process related to asymmetric division, where daughter cells proximal to the immunological synapse have an “effector phenotype” (FSC ^High^, SSC ^High^, CD62^Low^, CD8 ^High^, CD69 ^High^, CD43 ^High^, CD25 ^High^, CD44 ^High^) and express IFN-γ mRNA. In contrast, distal cells have an opposite phenotype, resembling a memory phenotype (61). Interestingly, our CD4^+^ and CD8^+^ SN^Bright^ and SN^-/Dim^ cells resemble such phenotypes, suggesting that Sial α2,6 expression could be a helpful marker to distinguish these populations using a single direct immunofluorescence.

These new described states open the door for new approaches to analyze T cell biology but also remind us that glycosylations are a fundamental path to further understanding how T cells adopt each of the roles they play, like becoming memory cells, getting activated, anergic, polarized, regulated or targeted for death. After all, the theoretical number of glycan determinants in the human glycome is approximately 7000 (62), with over 80 different glycan-binding proteins (63, 64). Finally, the potential clinical use of identifying active cells migrating to the infection/inflammation site by adding a single glycophenotypic marker provides novel approximations to patient monitoring, antigen discovery, and immunotherapy in infectious, autoimmune, or cancerous contexts.

## Data Availability

The raw data supporting the conclusions of this article will be made available by the authors, without undue reservation.
