# Supplementary material for: α2,6 sialylation distinguishes a novel active state in CD4+ and CD8+ cells during acute Toxoplasma gondii infection
Source: Front Immunol. 2024 Aug 26;15:1429302. doi: 10.3389/fimmu.2024.1429302 (PMC11381403; doi:10.3389/fimmu.2024.1429302)
Supplement: Supplementary file 1 [file Image1.pdf]

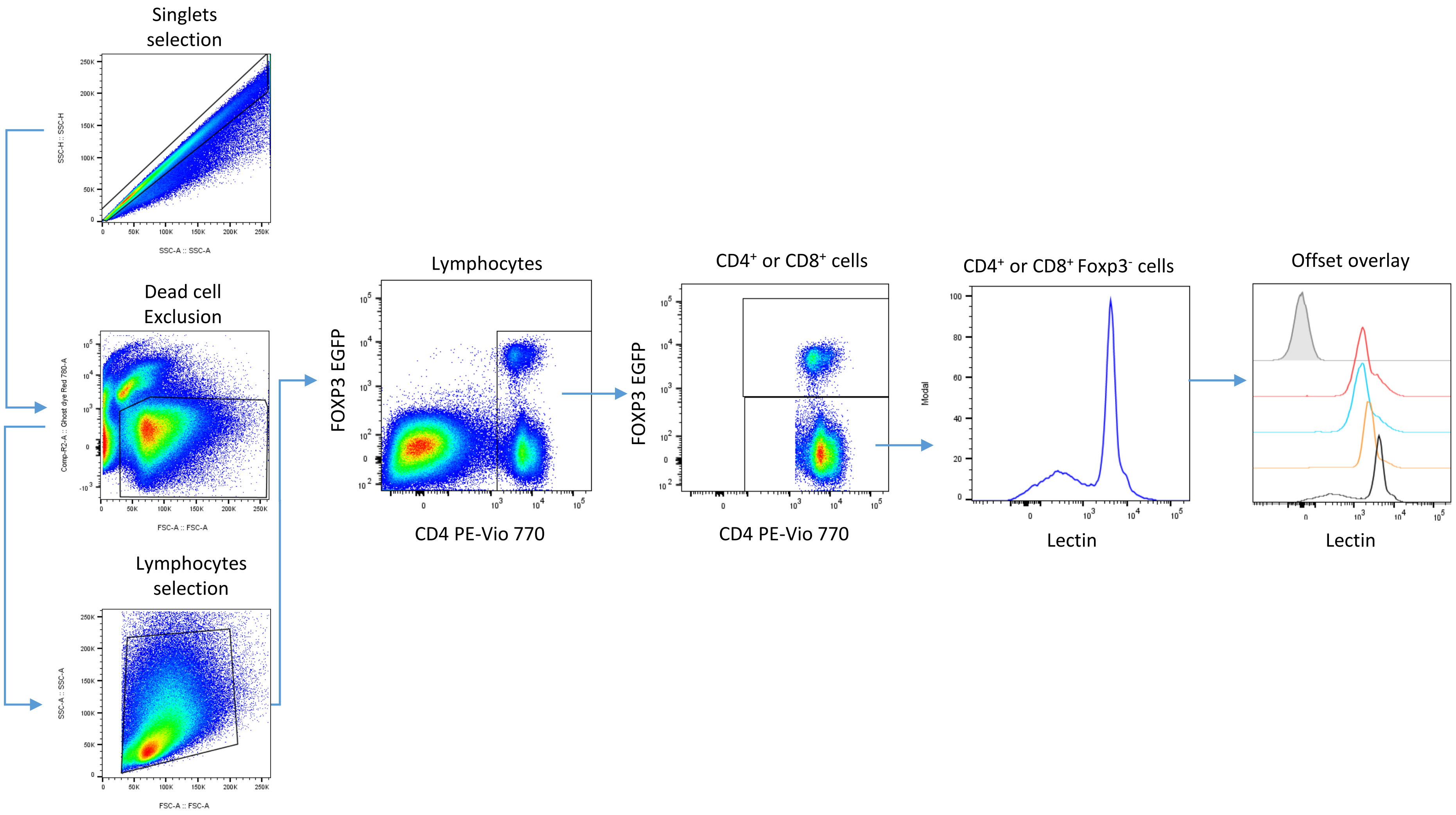

Supplementary Fig. 1

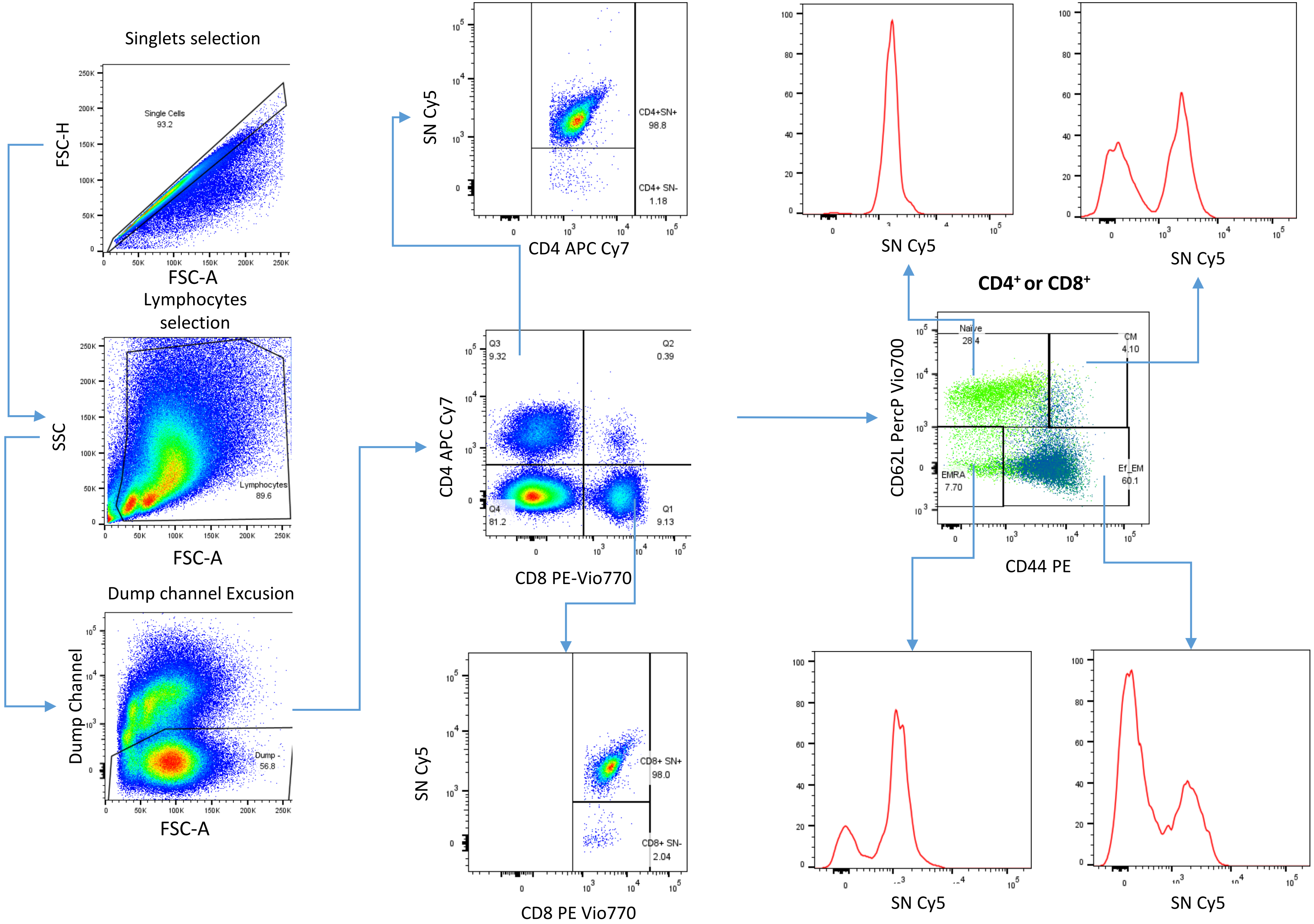

Supplementary Fig. 2

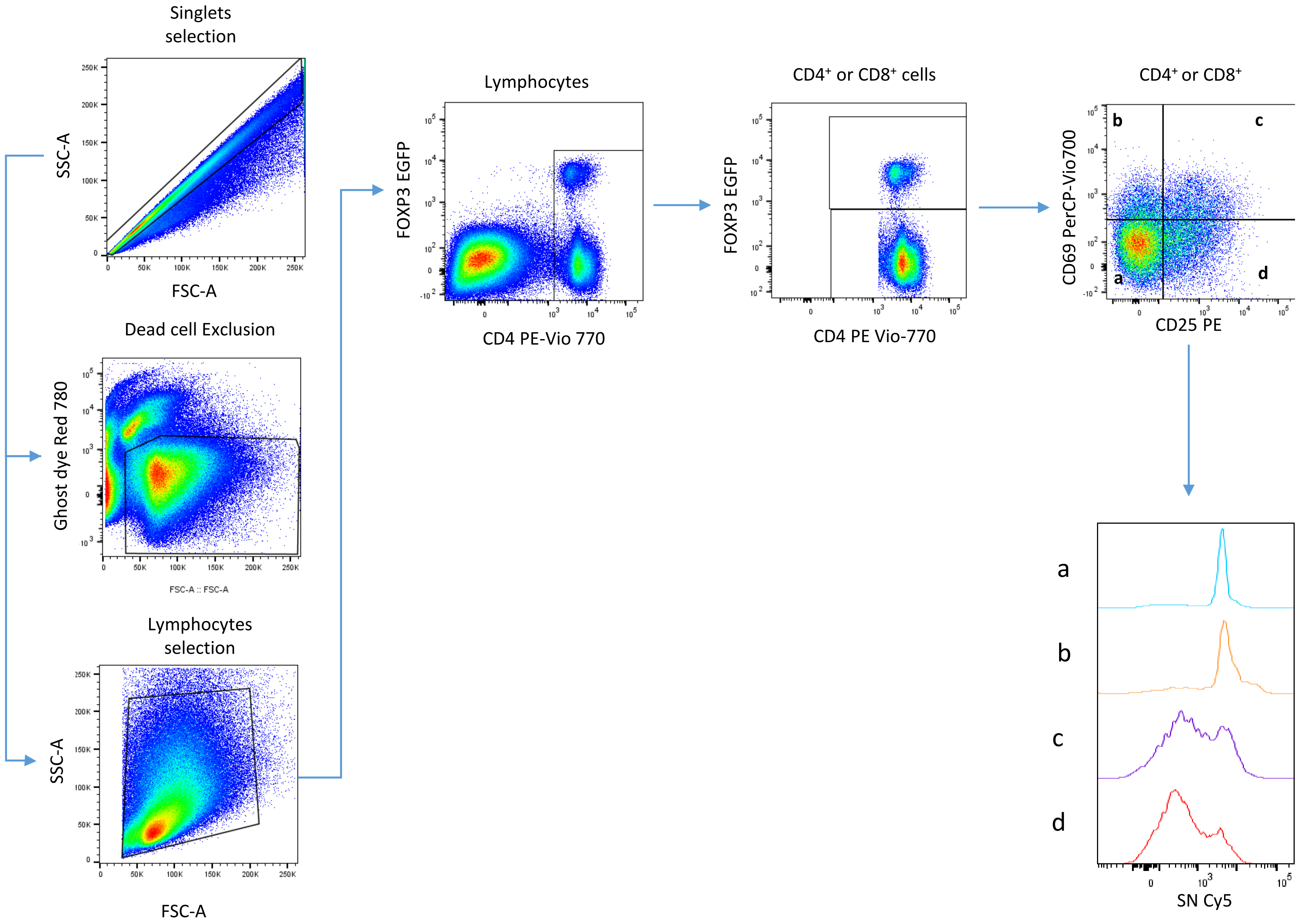

Supplementary Fig. 3

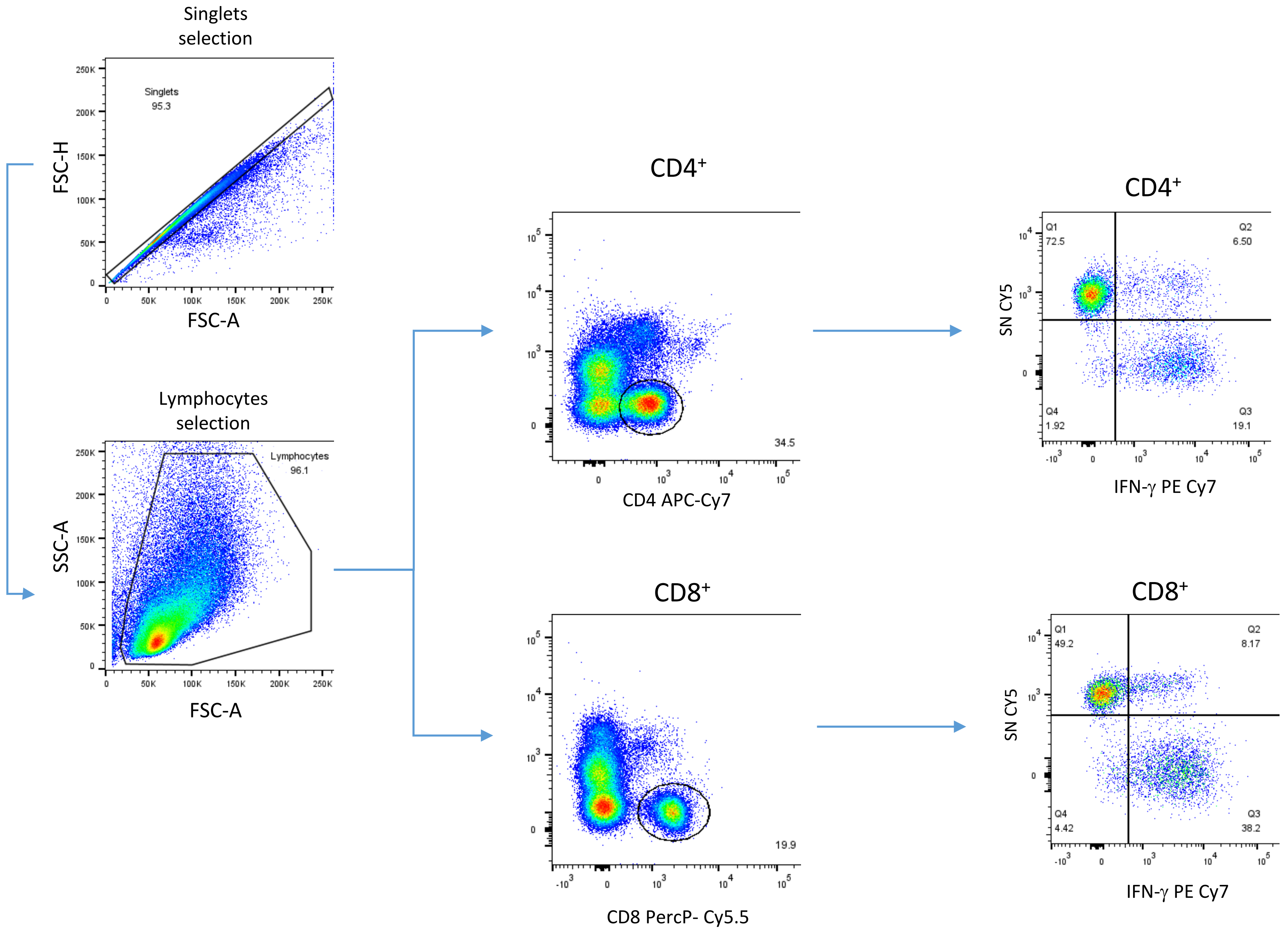

Supplementary Fig. 4

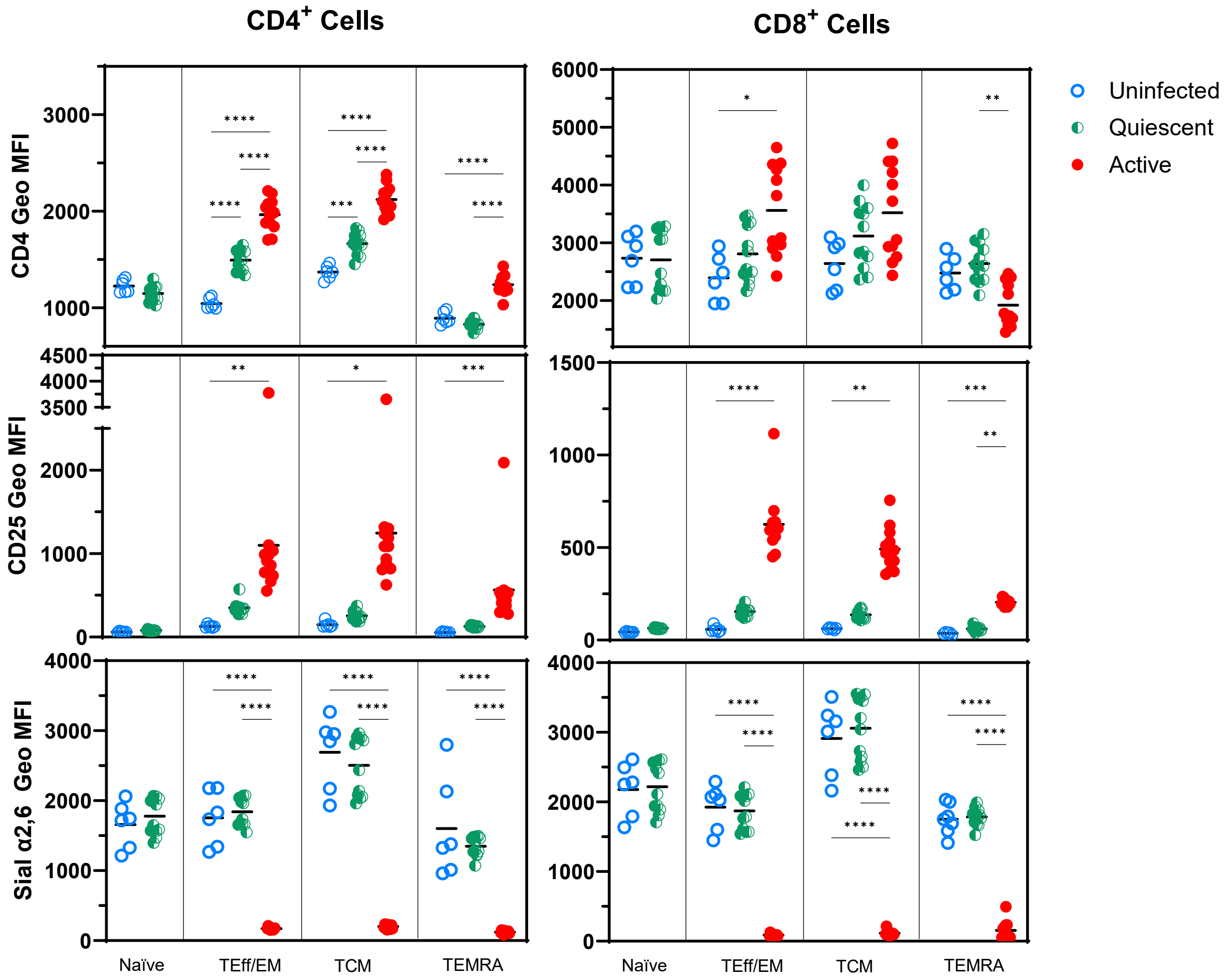

Supplementary Fig. 5

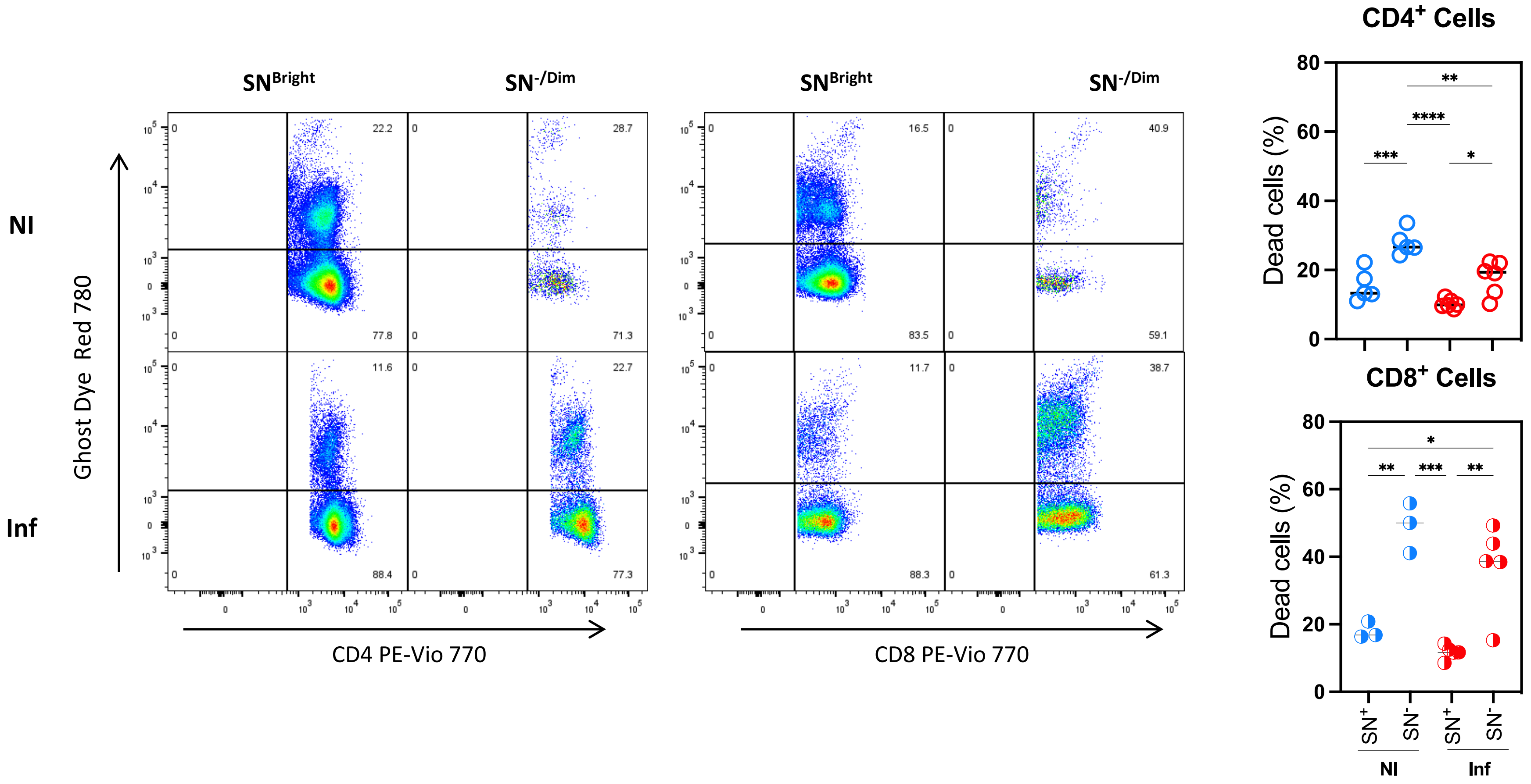

Supplementary Fig. 6. Dead cells are mainly Sial α2,6. Mouse splenocytes obtained at 10 dpi were stained using the panels indicated in Suppl Table 1 and analyzed by flow cytometry. After singlet selection, lymphocytes were selected by their FSC and SSC characteristics, $CD4^+$ $Foxp3^-$ or $CD8^+$ $Foxp3^-$ cells were selected, subgated according to their SN binding level and Ghost Dye Red 780 incorporation was determined. (A) Representative dot plots are shown for viability detection of SN^{Bright} and $SN^{-/Dim}$ within $CD4^+$ and $CD8^+$ cells at 10 dpi (Inf) and from uninfected (NI) mice. The percentage of dead cells within SN^{Bright} and $SN^{-/Dim}$ cells from uninfected (NI, blue) and infected (Inf, red) mice within $CD4^+$ (○) and $CD8^+$ (◐) lymphocytes is depicted. Statistical analysis was performed using one-way ANOVA with Bonferroni's multiple comparison test, *p<0.05, **p<0.001, ***p<0.005, ****p<0.0001.
