# Supplementary material for: α2,6 sialylation distinguishes a novel active state in CD4+ and CD8+ cells during acute Toxoplasma gondii infection
Source: Front Immunol. 2024 Aug 26;15:1429302. doi: 10.3389/fimmu.2024.1429302 (PMC11381403; doi:10.3389/fimmu.2024.1429302)
Supplement: Supplementary file 2 [file Table1.docx]

Supplementary Material

| **Channel** | **Panel 1**  **Figure 1, 2 and 4** | **Panel 2**  **Figures 3, 7 and 8** | **Panel 3**  **Figures 5 and 6** |
| --- | --- | --- | --- |
| **V1** | ALL Biot- Strp BV 421  or  MAL II Biot-Strp BV421 | CD25 BV 421 | IL-2 eFluor 450 |
| **B1** | Foxp3 EGFP | Dump Channel:  -Foxp3 EGFP  -CD16/32 FITC  -Zombie Green | Dump Channel:  -Foxp3 EGFP  -CD16/32 FITC  -Zombie Green |
| **B2** | CD25 PE | CD44 PE | IL-10 PE |
| **B3** | CD69 PercP-Vio700 | CD62L PercP-Vio700 | CD8 PercP-Cy5.5 |
| **B4** | CD4 PE-Vio770  or CD8 PE-Vio770 | CD8 PE-Vio770 | IFN-γ PE-Cy7 |
| **R1** | PNA Cy5 or  SN Cy5 | SN Cy5 | SN Cy5 |
| **R2** | Ghost Dye Red 780 | CD4 APC-Cy7 | CD4 APC-Cy7 |

**Supplementary Table 1**. Flow cytometry panels used for splenocytes staining. Samples were acquired in a MACSQuant cytometer, with the configuration depicted herein.

**Supplementary Table 2. Glycophenotype Index**

| **Glycophenotype** | **CD/TF Markers** | **Lectin Marker** | **Lectin Origin** | **Rough Sugar specificity** |
| --- | --- | --- | --- | --- |
| **CD4^+^ PNA^+^** | CD4^+^ Foxp3^-^ | PNA^+^ | Peanut  *Arachis hyggaea* | Galβ1,3GalNAc α1, O-Ser/Thr |
| **CD4^+^ PNA^-^** | CD4^+^ Foxp3^-^ | PNA^-^ |  |  |
| **CD8^+^ PNA^+^** | CD8^+^ Foxp3^-^ | PNA^+^ |  |  |
| **CD8^+^ PNA^-^** | CD8^+^ Foxp3^-^ | PNA^-^ |  |  |
|  | | | | |
| **CD4^+^ ALL^+^** | CD4^+^ Foxp3^-^ | ALL^+^ | *Amaranthus leucocarpus* | -Galβ1,3GalNAc α1, O-Ser/Thr  -GalNAc α1, O-Ser/Thr  Recognition occurs despite the presence of sialic acid in the structure |
| **CD4^+^ ALL^-^** | CD4^+^ Foxp3^-^ | ALL^-^ |  |  |
| **CD8^+^ ALL^+^** | CD8^+^ Foxp3^-^ | ALL^+^ |  |  |
| **CD8^+^ ALL^-^** | CD8^+^ Foxp3^-^ | ALL^-^ |  |  |
|  | | | | |
| **CD4^+^ MAL II^+^** | CD4^+^ Foxp3^-^ | MAL II^+^ | *Maackia amurensis* | Sial(α2,3)Galβ3GalNAc |
| **CD4^+^ MAL II^-^** | CD4^+^ Foxp3^-^ | MAL II^-^ |  |  |
| **CD8^+^ MAL II^+^** | CD8^+^ Foxp3^-^ | MAL II^+^ |  |  |
| **CD8^+^ MAL II^-^** | CD8^+^ Foxp3^-^ | MAL II^-^ |  |  |
|  | | | | |
| **CD4^+^ SN^Bright^** | CD4^+^ Foxp3^-^ | SN^Bright^ | *Sambucus nigra* | Sial(α2,6)Galβ3GalNAc |
| **CD4^+^ SN^-/Dim^** | CD4^+^ Foxp3^-^ | SN^-/Dim^ |  |  |
|  | | |  |  |
| **Naïve CD4^+^ SN^Bright^** | CD4^+^ Foxp3^-^ CD62L^Bright^ CD44^-/Low^ | SN^Bright^ |  |  |
| **Ef/EM CD4^+^ SN^Bright^** | CD4^+^ Foxp3^-^ CD62L^High^ CD44^High^ | SN^Bright^ |  |  |
| **CM CD4^+^ SN^Bright^** | CD4^+^ Foxp3^-^ CD62L^-/Low^ CD44^High^ | SN^Bright^ |  |  |
| **TEMRA CD4^+^ SN^Bright^** | CD4^+^ Foxp3^-^ CD62L^-^ CD44^Low/Int^ | SN^Bright^ |  |  |
|  | | |  |  |
| **Naïve CD4^+^ SN^-/Dim^** | CD4^+^ Foxp3^-^ CD62L^Bright^ CD44^-/Low^ | SN^-/Dim^ |  |  |
| **Ef/EM CD4^+^ SN^-/Dim^** | CD4^+^ Foxp3^-^ CD62L^High^ CD44^High^ | SN^-/Dim^ |  |  |
| **CM CD4^+^ SN^-/Dim^** | CD4^+^ Foxp3^-^ CD62L^-/Low^ CD44^High^ | SN^-/Dim^ |  |  |
| **TEMRA CD4^+^ SN^-/Dim^** | CD4^+^ Foxp3^-^ CD62L^-^ CD44^Low/Int^ | SN^-/Dim^ |  |  |
|  | | |  |  |
| **CD8^+^ SN^Bright^** | CD8^+^ Foxp3^-^ | SN^Bright^ |  |  |
| **CD8^+^ SN^-/Dim^** | CD8^+^ Foxp3^-^ | SN^-/Dim^ |  |  |
|  | | |  |  |
| **Naïve CD8^+^ SN^Bright^** | CD8^+^ Foxp3^-^ CD62L^Bright^ CD44^-/Low^ | SN^Bright^ |  |  |
| **Ef/EM CD8^+^ SN^Bright^** | CD8^+^ Foxp3^-^ CD62L^High^ CD44^High^ | SN^Bright^ |  |  |
| **CM CD8^+^ SN^Bright^** | CD8^+^ Foxp3^-^ CD62L^-/Low^ CD44^High^ | SN^Bright^ |  |  |
| **TEMRA CD8^+^ SN^Bright^** | CD8^+^ Foxp3^-^ CD62L^-^ CD44^Low/Int^ | SN^Bright^ |  |  |
|  | | |  |  |
| **Naïve CD8^+^ SN^-/Dim^** | CD8^+^ Foxp3^-^ CD62L^Bright^ CD44^-/Low^ | SN^-/Dim^ |  |  |
| **Ef/EM CD8^+^ SN^-/Dim^** | CD8^+^ Foxp3^-^ CD62L^High^ CD44^High^ | SN^-/Dim^ |  |  |
| **CM CD8^+^ SN^-/Dim^** | CD8^+^ Foxp3^-^ CD62L^-/Low^ CD44^High^ | SN^-/Dim^ |  |  |
| **TEMRA CD8^+^ SN^-/Dim^** | CD8^+^ Foxp3^-^ CD62L^-^ CD44^Low/Int^ | SN^-/Dim^ |  |  |
